# 

**Published:** 1858-09

**Authors:** 


### LIST OF COLLABORATORS.

---

S. G. Armor, M.D., late Professor of Pathology and Clinical Medicine, Missouri Med. College.
John L. Atlee, M.D., late President Pennsylvania State Medical Society.
Walter F. Atlee, M.D., Philadelphia.
Robert G. Barclay, M.D., Jerusalem, Syria.
John Bell, M.D., Philadelphia, formerly Professor of Medicine, Medical College of Ohio.
T. S. Bell, M.D., Professor of Medicine, University of Louisville.
S. M. Bemiss, M.D., Professor of Clinical Medicine, University of Louisville.
L. P. Blackburn, M.D., New Orleans, La.
George C. Blackie, M.D., Professor of Botany, University of Nashville.
G. C. Blackman, M.D., Professor of Surgery, Medical College of Ohio.
W. S. Bowen, M.D., New York.
J. L. Bobbs, M.D., formerly Professor of Anatomy, Indianapolis.
W. M. Boling, M.D., Montgomery, Ala., formerly Professor of Midwifery, Transylvania University, Lexington, Ky.
N. Bozeman, M.D., Montgomery, Ala.
J. T. Bradford, M.D., Augusta, Ky.
John H. Brinton, M.D., Lecturer on Operative Surgery, Philadelphia.
W. S. Chipley, M.D., Professor of Medicine, Transylvania University.
A. Clapp, M.D., New Albany, Ia.
D. B. Cliffe, M.D., Franklin, Tenn.
J. Da Costa, M.D., Lecturer on the Practice of Medicine at the Philad. Med. Association.
Samuel H. Dickson, M.D., Professor of Medicine, Jefferson Medical College.
M. Dupierris, M.D., Havana, Cuba.
W. F. Edgar, M.D., United States Army.
S. C. Farrar, M.D., Jackson, Miss.
C. S. Fenner, M.D., Memphis, Tenn.
Austin Flint, M.D., Professor of Clinical Medicine, Auscultation and Percussion, New Orleans School of Medicine.
Charles Frick, M.D., Baltimore.
W. Y. Gadbury, M.D., Benton, Miss.
O. C. Gibbs, M.D., Chautauque County, New York.
Dr. J. P. Hall, Glasgow, Ky.
John Hardin, M.D., Professor of Obstetrics, Kentucky School of Medicine, Louisville.

EDWARD HARTSHORNE, M.D., One of the Surgeons to Wills Hospital for Diseases of the Eye, Philadelphia.

E. B. HASKINS, M.D., Professor of Chemistry, Shelby Medical College, Nashville, Tenn.

C. A. HENTZ, M.D., Marianna, Florida.

ADDINELL HEWSON, M.D., One of the Surgeons to Wills Hospital for Diseases of the Eye, Philadelphia.

SAMUEL L. HOLLINGSWORTH, M.D., late Editor of the Medical Examiner, Philadelphia.

WILLIAM A. HAMMOND, M.D., United States Army.

WILSON JEWELL, M.D., Philadelphia, President of the Quarantine and Sanitary Convention.

WM. V. KEATING, M.D., Lecturer on Obstetrics and the Diseases of Women, at the Philadelphia Medical Association.

JOHN G. KERR, M.D., Canton, China.

G. A. KUNKLER, M.D., Madison, Ia.

BEN. LOGAN, M.D., Shreveport, La.

A. LOPEZ, M.D., Mobile, Ala., formerly President of the Alabama State Medical Society.

GEORGE R. MOREHOUSE, M.D., Philadelphia.

S. WEIR MITCHELL, M.D., Lecturer on Physiology at the Philad. Med. Association.

B. I. RAPHAEL, M.D., New York.

J. C. REEVE, M.D., Dayton, Ohio.

L. C. RIVES, M.D., formerly Professor of Midwifery, Medical College of Ohio.

J. LAWRENCE SMITH, M.D., Professor of Chemistry, University of Louisville.

W. C. SNEED, M.D., Frankfort, Ky., late President Kentucky State Medical Society.

C. H. SPILMAN, M.D., Harrodsburg, Ky., late President of Kentucky State Medical Society.

GEO. SUTTON, M.D., Aurora, Ia.

W. L. SUTTON, M.D., Georgetown, Ky., formerly President Kentucky State Medical Society.

HORATIO R. STORER, M.D., formerly one of the Physicians to the Boston Lying-in Hospital, Boston, Mass.

S. THOMPSON, M.D., Albion, Ill., late President of the Illinois State Medical Society.

E. WILLIAMS, M.D., Cincinnati, Ohio.

### Bi-Monthly Bibliographical Record.

AMERICAN.

A Treatise on the Practice of Medicine. By George B. Wood, M.D., Professor of the Theory and Practice of Medicine in the University of Pennsylvania, etc. etc. In two volumes. Fifth edition. 8vo., pp. 1792. J. B. Lippincott & Co.: Philadelphia, 1858. (From the author.)

Ventilation in American Dwellings, etc. By David Boswell Reid, M.D., F.R.S.E., etc. etc., and Elisha Harris, M.D. 8vo., pp. 124. New York: Willey & Halstead, 1858. (From the publishers.)

Pathology and Treatment of the Paralysis of Muscular Motion. By J. P. Batchelder, M.D. 8vo., pp. 62. New York, 1858. (From the author.)

On Intra-Capsular Fracture of Cervix Femoris with Bony Union; and an Interesting case of Urinary Calculi. By Alden March, M.D. Pamphlet, 8vo., pp. 26. Albany, 1858.

An Essay on the Pathology and Therapeutics of Scarlet Fever. By Caspar Morris, M.D., etc. etc. Second edition. 8vo., pp. 189. Lindsay & Blakiston: Philadelphia, 1858. (From the publishers.)

Transactions of the Twenty-Ninth Annual Meeting of the Tennessee State Medical Society; held at Nashville. 8vo., pp. 43. 1858.

FOREIGN.

Handbuch der Chirurgischen Anatomie. Von F. Führer, M.D., Prosector an der Anatomischen Lehranstalt in Hamburg, etc. 8vo., pp. 1205. George Reimer: Berlin, 1857.

A Manual of Psycological Medicine. By John C. Bucknill, M.D., and Daniel H. Tuke, M.D. 8vo., pp. 536. Republished by Blanchard & Lea: Philadelphia, 1858. (From the publishers.)

Notes on the Surgery of the Crimean War; with Remarks on Gun-Shot Wounds. By George H. B. Macleod, M.D., F.R.C.S., Edinb., etc. 8vo. Churchill: London, 1858.

An Essay on the Ophthalmoscope; its Management and Use in the Exploration of the Internal Eye Diseases. By Jabez Hogg. Churchill: London, 1858.

On Localized Galvanism; Applied to the Treatment of Paralysis and Muscular Contractions. By Richard M. Lawrence, M.D., Ophthalmic Surgeon to the Great Northern Hospital. Renshaw: London, 1858.

Stricture of the Urethra; its Complications, Symptoms, and Treatment; with Cases, illustrative of a Mode of Treating its more Intractable Forms. By Robert Wade, F.R.C.S., etc. 8vo. Churchill: London, 1858.

Strangulated Hernia; a Clinical Memoir, with the Author's Practice, etc. By George Macilwain, F.R.C.S., formerly Surgeon to the City of London Truss Society. Renshaw: London, 1858.

Chronic Phthisis, the Principles of Treatment of. By Edward Smith, M.D., L.L.B., Lond., etc. 8vo. Longman & Co.: London, 1858.

Essay on the Pathology of the Blood and its Containing Vessels. By Thomas A. Wise, M.D., F.R.S.E., etc. etc. Demy 8vo., pp. 388. Longman & Co.: London, 1858.

An Essay on Physiological Psychology. By Robert Drum, F.R.C.S., Eng. 8vo. Churchill: London, 1858.

Inversio Vesicæ Urinariæ og Luxationes Femorum Congenitæ hos sommæ Individ, iagttagne af Lektor Voss. Hermed 2de farvetrykte Plancher. 4to., pp. 25. Christiana: Brogger & Christie, 1857. (From the author.)

The Human Mind in Relation with the Brain and Nervous System. By Daniel Noble, M.D. Post 8vo. Churchill: London, 1858.

The Medical and Legal Relations of Madness. By Joshua Burgess, M.D. 8vo. Churchill: London, 1858.

The Complete Hand-Book of Obstetric Surgery. By Charles Clay, M.D., etc. Fcap. 8vo. Renshaw: London, 1858.

A Treatise on the Pathology of the Urine, including a Complete Guide to its Analysis. By J. L. W. Thudichum, M.D. 8vo. Churchill: London, 1858.

A Manual of Obstetrics. By W. Tyler Smith, M.D., Lecturer on Midwifery at St. Mary's Hospital. Fcap. 8vo. Churchill: London, 1858.

Observations on Naval Hygiene and Scurvy, more particularly as the latter appeared during a Polar Voyage. By Alex. Armstrong, M.D., R.M. 8vo. Churchill: London, 1858.

Epilepsy and other Convulsive Affections; their Pathology and Treatment. By Charles Bland Radcliffe, M.D., Physician to the Westminster Hospital. Second edition. Post 8vo. Churchill: London, 1858. (From the author.)

On Diabetes and its Successful Treatment. By John M. Camplin, M.D., F.L.S. Fcap. 8vo. Churchill: London, 1858.

Regional Anatomy. By M. W. Hilles, formerly Lecturer at the Westminster School of Medicine, etc. 16mo., pp. 184. Renshaw: London, 1857

